# Serum amylase as a prognostic marker of organophosphate poisoning

**DOI:** 10.5249/jivr.vo113i2.1632

**Published:** 2021-07

**Authors:** Mehdi Zobeiri

**Affiliations:** ^a^ Department of Internal Medicine, Imam Reza Hospital, Kermanshah University of Medical Sciences, Kermanshah, Iran.

**Keywords:** Organophosphate, Intoxication, Amylase, Prognosis

## Abstract

**Background:**

Organophosphate (OP) insecticides are important compounds as the most probable common cause of acute poisonings in developing countries. OP intoxication often presents as medical emergencies, and its related morbidity and mortality have not decreased despite major advances in critical care. This study aims to determine the impact of serum amylase level for estimation of prognosis in patients with acute OP poisoning.

**Methods:**

This observational case-control study was done during two years on 332 consecutive patients with acute OP poisoning. Clinical and demographic data, serum amylase level on early admission time, morbidity, and outcome were determined. Data were analyzed in the form of a frequency distribution table by using SPSS 11.0 version software.

**Results:**

The mean age of patients with acute OP poisoning was 28.9 ± 23.95 with slightly female dominance. All patients were intoxicated via the gastrointestinal route. The mean amylase level of patients with deterioration of mental status, tachycardia, ICU admission, and death was significantly higher.

**Conclusions:**

Among patients with OP poisoning, higher serum amylase than normal was associated with severe clinical course and increased risk for mortality. Determination of serum amylase can be effective in the quick prediction of the outcome.

## Introduction

Organophosphates (OP) have become the most widely used agricultural insecticide worldwide. Suicidal attempt with these easily accessible agents is a major problem in developing countries.^1^ These agents are also used in chemical warfare as nerve agents.^2^ OP is absorbed rapidly via the oral, respiratory, or transdermal routes, and has a high level of morbidity and mortality, especially in developing countries.^3,4^ The mortality rate of self-poisoning in developing countries is 10–20%, mainly caused by respiratory insufficiency secondary to central depression of respiration, muscle weakness, and/or direct lung effects by bronchospasm and bronchorrhea.^5,6^ Several syndromes are associated with OP poisonings, like acute cholinergic crises, intermediate syndrome (IMS) which can proceed from ~20% of first, and OP-induced delayed neuropathy.^4^ Because of high mortality risk, both the acute cholinergic crises and the intermediate syndrome are best managed in an intensive care unit, unless the poisoning has been very mild.^4^ The principal pharmacological action is inhibition of acetylcholinesterase with acetylcholine accumulation at all ganglia in the autonomic nervous system and many synapses in the brain, skeletal neuromuscular junctions, some postganglionic nerve endings of the sympathetic nervous system and nerve of the adrenal medulla, leading to overstimulation of acetylcholine receptors.^3,4^ Currently, this view has challenged that only inhibition of cholinesterase cannot account for the wide range of impairments that have been reported following OP poisoning.^4^ Manifestation of OP poisoning is categorized as the muscarinic, nicotinic, and central nervous system. Overstimulation of muscarinic receptor present with parasympathetic excitement, including miosis, bradycardia, and bronchorrhea. The nicotinic reaction consists of muscle fasciculation, cramping, and weakness, while loss of consciousness, respiratory depression, and seizures are due to central nervous system effects. Diagnosis mainly is based on clinical signs.^7^ Measurement of plasma cholinesterase is the most distinct test of OP poisoning.^8^ Early antagonism of OP poisoning as evidence suggested, should be accompanied by better outcomes.^9^ Increased serum amylase is a well-documented biochemical derangement of OP poisoning which may be due to parasympathetic overstimulation of the pancreas.^10^ The present study was performed to determine the impact of serum amylase level in estimation of severity considered by certain parameters such as respiratory failure, coma, hemodynamic disturbances, and prognosis in patients with acute OP poisoning. 

## Materials and Methods 

This observational case-control study was done during two years on 332 consecutive patients with acute OP poisoning in intensive care and toxicological unit. Patients who enrolled in this study were diagnosed with OP poisoning through history by themselves or their companion and associated clinical features. Plasma cholinesterase was not measured. Serum amylase without distinction of pancreatic or salivary type on early admission time was determined by a photometric assay using a quantitative diagnostic kit of pars Azmoun company (Tehran, Iran), then demographic data, clinical symptoms (gastrointestinal and respiratory tract symptoms), and signs (pulse rate, respiratory rate, blood pressure, Glasgow Coma Scale (GCS), need for mechanical ventilation, duration of hospital stay, mortality and received amount of atropine) were determined and collected by pre-designed questionnaire. Definition of hypotension is systolic blood pressure of 90 mm Hg or less, hypertension above 140 mm Hg, bradycardia by pulse rate less than 60 beats per minute, and tachycardia by a heart rate of 100 beats per minute or higher. Definition of coma is GCS of 9 or less and hyperamylasemia is a serum concentration more than 100 units per liter (U/L) (normal <100 U/L). All patients received pralidoxime by protocol (2 gr stat and 2 gr q 8 h up to 3 days) and atropine based on symptoms and signs mainly crackles, excess body secretions (saliva, sweat). Data were analyzed in the form of a frequency distribution table using SPSS 11.0 version software. Continuous variables were expressed as means. Quantitative variables were analyzed with Pearson's coefficient and for comparison of the incidence of the complications chi-square test was used. A P-value of ≤ 0.05 was considered statistically significant. 

## Results

Of 332 patients, 179 (53.9%) were female and 153 (46.1%) were males. Their mean age was 28.9± 13.95 years. All intoxication was by acute ingestions of a single OP with the intention of suicide. In the identified cases the most commonly used toxins were chlorpyrifos, diazinon, and dimethoate. Most frequent clinical features were tachycardia (73.1%), gastrointestinal symptoms (nausea, vomiting, abdominal pain) (29.7%), respiratory problems (crackles, tachypnea) (14.15%), Confusion and coma (14.8%), and hypotension (1.2%) (Table 1). No clinically significant bradycardia has been recorded. The mean hospital stay was 2.2± 2.28 days and 15.4% of patients were admitted to the intensive care unit. The mean serum amylase level in all patients was 114.5± 103.24 U/L and an average of 350.94±164.7 mg atropine per patient was administered. Mortality rate for all 322 OP exposures was 2.1%. The mean amylase level in patients with confusion or coma and tachycardia were significantly higher (p <0.05) (Table 1).

**Table 1:** Frequency of clinical variables and relation with serum amylase. (P-value ≤ 0.05 considered significant)

Groups/Clinical Variable	Frequency	Mean amylase in patients with clinical variables	Mean amylase in patients without clinical variables	P value
**Tachycardia**	73%	159.49	107.30	0.029
**Gastrointestinal symptoms**	29%	110.65	115.61	0.691
**Coma & Confusion**	14.8%	194.95	103.64	0.015
**Respiratory sign**	14.1%	209.21	101.40	0.087
**Fever **	3%	109.10	114.69	0.866
**Hypotension**	1.2%	102.5	114.67	0.815

Non survived patients and patients who need ICU admission had higher amylase level (p<0.05) (Table 2,3). 

**Table 2:** Correlation of serum amylase and survival. (P-value ≤ 0.05 considered significant)

Survival	Survived (%)	Non survived(%)	P value
Amylase	7 (2.1%)	325 (97.9)	
**Mean serum amylase level(U/L)**	112.78	195.29	0.036

**Table 3:** Correlation of serum amylase and ICU admission. (P-value ≤ 0.05 considered significant)

ICU admission	With ICU admission (%)	whithout ICU admission (%)	P value
Amylase	51 (15.4%)	281 (84.6%)	
**Mean serum amylase level(U/L)**	176.76	103.22	0.012

ICU admission has been mostly for respiratory support.

## Discussion

In this study rise in plasma amylase, more than 60% above the normal upper limit on the admission time was associated with clinical severity and mortality following OP poisoning. In other studies, this correlation was confirmed and recommended check of plasma amylase in all of the OP poisoning for prediction of severity of clinical sign, respiratory failure, shock, and coma.^11-14^ Hyperamylasemia is frequent in severe OP poisoning and some authors considered it as a marker of OP poisoning.^13-15^ Acute pancreatitis in adults with OP poisoning is estimated to be 12%, but hyperamylasemia because of low sensitivity and specificity is not a valid parameter in the diagnosis of OP-induced pancreatitis.^12-16^ So, serum lipase may be useful for the early diagnosis of pancreatitis.^13^ Tachycardia, as a most common clinical manifestation and associated with increased amylase, is a less frequent nicotinic sign (by stimulation of sympathetic ganglia), but here seems to arise mainly from atropine administration. The amount of received atropine is based on the continued stability of muscarinic signs and symptoms. Tachycardia, which is a symptom of atropinization, confirms adequate atropine intake. Therefore, it can be claimed that the severity and duration of muscarinic manifestation are related to prognosis. After justifying tachycardi, gastrointestinal symptoms are the most frequent presentation.^4^

Oral ingestion of OP pesticides in a suicidal attempt is a major health problem especially in developing countries.^17^ Diagnosis and aggressive management of acute poisoning with these lethal substances are required for decreasing morbidity and mortality.^18,19^ Time of antidote administration seems to be a key factor in patient´s outcome.^20^ Life-threatening intoxication is characterized by altered consciousness, seizure, urinary incontinence, and respiratory suppression. Respiratory failure is a most common complication and cause of death.^18^ In this study high amylase level in admission time predict a higher probability of having morbidity (ICU admission, confusion or coma, need more atropine for symptoms relief) and mortality. Multiple systems have been introduced for the prediction of outcomes in OP poisoning which can be based on clinical or laboratory data. Noshad H et al. showed a mortality rate of patients with mechanical ventilation of 50%, despite 11.7% without mechanical ventilation.^19^ Liu JH et al. showed 50% mortality of acute OP poisoning with respiratory acidosis and 25% with metabolic acidosis, thus the prediction of the outcome in patients with acute OP poisoning can be determined by quick acid-base interpretation before hospitalization.^21^ Respiratory acidosis, ICU admission, and mechanical ventilation are intertwined and represent one concept. It was not possible to measure plasma cholinesterase (PCE) that decreased by OP induced acetylcholinesterase inhibition. In Lin C-L et al. and Goswamy R et al. studies, miosis, unconsciousness, bradycardia, fasciculation, and low PCE level were significant factors associated with respiratory failure and requirement of ventilatory support.^22,23^ Chaudhary SC et al. recommend quantification of PCE level at the admission time for categorization of OP poisoning severity and estimation of prognosis.^24^ Serum amylase showed a statistically significant negative correlation with PCE and the highest accuracy for severity assessment of OP poisoning then followed by keratin phosphokinase (CPK) and Lipase.^25^

**Acknowledgment**

This study was funded by Kermanshah University of Medical Sciences as a thesis research project. I wish to express my best appreciation to the personnel of emergency department and to all of those who supported me in any respect during the completion of this research.

## References

[1] Georgiadis G, Mavridis C, Belantis C, Zisis IE, Skamagkas I, Fragkiadoulaki I (2018). Nephrotoxicity issues of organophosphates. Toxicology.

[2] Rao AN, Patil A, Brodnik ZD, Liang Qiang, Rodrigo A España, Kimberly A Sullivan (2017). Pharmacologically increasing microtubule acetylation corrects stressexacerbated effects of organophosphates on neurons. Traffic.

[3] Davies JO, Eddleston M, Buckley NA (2008). Predicting outcome in acute organophosphorus poisoning with a poison severity score or the Glasgow coma scale. QJM.

[4] Jokanović M (2018). Neurotoxic effects of organophosphorus pesticides and possible association with neurodegenerative diseases in man: A review. Toxicology.

[5] Lorke DE, Petroianu GA (2019). Reversible cholinesterase inhibitors as pretreatment for exposure to organophosphates. A review. J Appl Toxicol.

[6] Eddleston M, Mohamed F, Davies JO, Eyer P, Worek F, Sheriff MH (2006). Respiratory failure in acute organophosphorus pesticide self-poisoning. QJM.

[7] Kumar A, Margekar SL, Margekar P, Margekar V (2018). Recent advances in Management of Organophosphate & Carbamate Poisoning. Indian J Med Res.

[8] Amanvermez R, Baydın A, Yardan T, Başol N, Günay M (2010). Emergency laboratory abnormalities in suicidal patients with acute organophosphate poisoning. Turk J Biochem.

[9] Bird SB, Gaspari RJ, Dickson EW (2003). Early death due to severe organophosphate poisoning is a centrally mediated process. Acad Emerg Med.

[10] Dagli AJ, Shaikh WA (1983). Pancretic involvement in malation anticholinesterase insecticide intoxication. A study of 75 case. Br J Clin Pract.

[11] Hou R, Zhang H, Chen H, Zhou Y, Long Y, Liu D (2019). Total pancreatic necrosis after organophosphate intoxication. Front Med.

[12] Moussa ME, Mohamed SA, Hilal MA, Elnabi MA, Zaki NA (2018). The Role of APACHE II, SOFA, Serum amylase and Lipase in Assessment of Severity and Outcome of Acute Organophosphorus Poisoning. Ain-Shams J Forensic Med.

[13] Hsiao CT, Yang CC, Deng JF, Bullard MJ, Liaw SJ (1996). Acute pancreatitis following organophosphate intoxication. J Toxicol Clin Toxicol.

[14] Lee WC, Yang CC, Deng JF, Wu ML, Ger J, Lin HC (1998). The clinical significance of hyperamylasemia in organophosphate poisoning. J Toxicol Clin Toxicol.

[15] Nagabhiru S (2020). A prospective study of serum amylase levels in acute organophosphorus poisoning and its relationship with its severity and outcome. J Assoc Physicians India.

[16] Singh S, Bhardwaj U, Verma SK, Bhalla A, Gill K (2007). Hyperamylasemia and acute pancreatitis following anticholinesterase poisoning. Hum Exp Toxicol.

[17] Lyu CP, Pei JR, Beseler LC, Li YL, Li JH, Ren M (2018). Case control study of impulsivity, aggression, pesticide exposure and suicide attempts using pesticides among farmers. Biomed Environ Sci.

[18] Sungur M, Guven M (2001). Intensive care management of organophosphate insecticide poisoning. Crit Care.

[19] Noshad H, Ansarin K, Ardalan MR, Ghaffari AR, Safa J, Nezami N (2007). Respiratory failure in organophosphate insecticide poisoning. Saudi Med J.

[20] Sivaganabalan R, Mohd Jamin Z, Loke KY, Z’aba N, Periathamby S (2018). Retrospective observational study of organophosphate poisoning in an urban Malaysian Hospital. J Clin Toxicol.

[21] Liu JH, Chou CY, Liu YL, Liao P-Y, Lin P-W, Lin H-H (2008). Acid-base interpretation can be the predictor of outcome among patients with acute organophosphate poisoning before hospitalization. Am J Emerg Med.

[22] Lin C-L, Yang C-T, Pan K-Y, Huang C-C (2004). Most common intoxication in nephrology ward organophosphate poisoning. Ren Fail.

[23] Goswamy R, Chaudhuri A, Mahashur AA (1994). Study of respiratory failure in organophosphate and carbamate poisoning. Heart Lung.

[24] Chaudhary SC, Singh K, Sawlani KK, Jain N, Vaish AK, Atam V (2013). Prognostic significance of estimation of pseudocholinesterase activity and role of pralidoxime therapy in organophosphorous poisoning. Toxicol Int.

[25] Sumathi ME, Kumar SH, Shashidhar KN, Takkalaki N (2014). Prognostic significance of various biochemical parameters in acute organophosphorus poisoning. Toxicol Int.

